# Exploring the effects of taurolidine on tumor weight and microvessel density in a murine model of osteosarcoma

**DOI:** 10.32604/or.2024.050907

**Published:** 2024-06-20

**Authors:** LISANNE K.A. NEIJENHUIS, LEUTA L. NAUMANN, SONIA A.M. FERKEL, SAMUEL J.S. RUBIN, STEPHAN ROGALLA

**Affiliations:** 1 Division of Gastroenterology and Hepatology, Department of Medicine, School of Medicine, Stanford University, Stanford, 94305, USA; 2 Department of Surgery, Leiden University Medical Center, Leiden, 2333 ZA, The Netherlands; 3 Centre for Human Drug Research, Leiden, 2333 CL, The Netherlands; 4 Department of General, Visceral, Vascular and Thoracic Surgery, Charité University Medicine Berlin, Berlin, 10117, Germany

**Keywords:** Osteosarcoma, Taurolidine, Cancer treatment, Chemotherapy, Murine models

## Abstract

**Background:**

Osteosarcoma is the most common malignant primary bone tumor. The prognosis for patients with disseminated disease remains very poor despite recent advancements in chemotherapy. Moreover, current treatment regimens bear a significant risk of serious side effects. Thus, there is an unmet clinical need for effective therapies with improved safety profiles. Taurolidine is an antibacterial agent that has been shown to induce cell death in different types of cancer cell lines.

**Methods:**

In this study, we examined both the antineoplastic and antiangiogenic effects of taurolidine in animal models of osteosarcoma. K7M2 murine osteosarcoma cells were injected, both intramuscular and intraperitoneal, into 60 BALB/c mice on day zero. Animals were then randomized to receive treatment with taurolidine 2% (800 mg/kg), taurolidine 1% (400 mg/kg), or NaCl 0.9% control for seven days by intravenous or intraperitoneal administration.

**Results:**

After 35 days, mice were euthanized, and the tumors were harvested for analysis. Eighteen mice were excluded from the analysis due to complications. Body weight was significantly lower in the 2% taurolidine intraperitoneal treatment group from day 9 to 21, consistent with elevated mortality in this group. Intraperitoneal tumor weight was significantly lower in the 1% (*p* = 0.003) and 2% (*p* = 0.006) intraperitoneal taurolidine treatment groups compared to the control. No antineoplastic effects were observed on intramuscular tumors or for intravenous administration of taurolidine. There were no significant differences in microvessel density or mitotic rate between treatment groups. Reduced body weight and elevated mortality in the 2% taurolidine intraperitoneal group suggest that the lower 1% dose is preferable.

**Conclusions:**

In conclusion, there is no evidence of antiangiogenic activity, and the antitumor effects of taurolidine on osteosarcoma observed in this study are limited. Moreover, its toxic profile grants further evaluation. Given these observations, further research is necessary to refine the use of taurolidine in osteosarcoma treatment.

## Introduction

Osteosarcoma is the most common malignant primary bone tumor and primarily affects children and adolescents. These tumors arise from primitive mesenchymal cells and typically develop in the distal femur, proximal tibia, and proximal humerus. Osteosarcoma is a highly aggressive tumor that metastasizes early to other parts of the body, specifically the lungs [1,2]. To grow and metastasize all cancers require development and maturation of blood vessels. This process is called angiogenesis, and drugs to alter this process are used in clinical care already with mixed success [3]. Osteosarcomas show pronounced vascularization, and several studies demonstrated strong correlations between high levels of vascular endothelial growth factor (VEGF) and tumor progression, metastasis, and poor prognosis; thus, anti-angiogenetic therapies may be particularly successful [4–8].

The introduction of chemotherapy radically improved survival rates. Current standard of care consists of surgical resection of the primary tumor and both neoadjuvant and adjuvant multi-agent chemotherapy. This approach has led to a 5-year survival rate of 70% in patients with localized disease. However, the survival of patients with metastatic disease remains very poor, with a 5-year survival rate of 20% [1,9]. The currently preferred chemotherapy regimen consisting of methotrexate, doxorubicin, and cisplatin is associated with numerous serious side effects, such as hearing loss, nephrotoxicity, cardiotoxicity, and secondary malignancies [10]. Therefore, there is a significant need for new options to treat osteosarcoma with less toxicity and similar or better efficacy.

Taurolidine (bis (1,1-dioxoperhydro-1,2,4-thiabiazin-4-yl) methane, or TRD) is a synthetically produced derivative of the amino acid taurine. The compound has bactericidal activity, targeting both aerobic and anaerobic bacteria [11,12]. In multiple European countries, TRD is authorized for the intraperitoneal treatment of peritonitis [13–15]. Moreover, taurolidine has exhibited a remarkable safety profile, being largely nontoxic and free of adverse effects since its introduction more than 40 years ago. Besides the anti-inflammatory properties of taurolidine, it has also been found to induce cancer cell death through multiple mechanisms, including augmentation of apoptosis, inhibition of angiogenesis and tumor adherence, downregulation of proinflammatory cytokines and endotoxin levels, and stimulation of the immune system in response to surgically induced trauma [16–18]. These mechanisms have been defined using multiple tumor cell lines both *in vitro* and *in vivo* [19–23].

In this study, we evaluate the effect of taurolidine on tumor growth and distribution in a murine model of osteosarcoma. Given established inhibitory effects of taurolidine on vascular endothelial growth factor (VEGF) and the significance of angiogenesis for osteosarcoma, we also assess the effect of taurolidine on microvessel density and mitotic rate by immunohistochemistry.

## Materials and Methods

### Cell lines and cell preparation

The murine osteosarcoma cell line K7M2 was purchased from the American Type Culture Collection (ATCC). Cells were cultured in Dulbecco’s MEM cell culture medium with L-glutamine (Biochrom AG, Berlin, Deutschland). The medium was supplemented with penicillin/streptomycin (5/500 mL; Biochrom AG) and 10% fetal calf serum (FCS; 50/500 mL; Biochrom AG). Cells were grown in an incubator at 37°C with 5% CO_2_.

In preparation for cell implantation, the medium was removed, and the cells were washed with phosphate buffered saline (PBS) solution. Afterwards, cells were detached by adding EDTA/trypsin and 5-min incubation at 37°C with 5% CO_2_. Then, 10 ml of pure medium was added, and the cells were resuspended. To remove trypsin, the cell suspension was centrifuged at 900 rpm for 7 min at 12°C and the supernatant was aspirated. To remove foreign antigens, the cell pellets were resuspended in pure medium without FCS. Cell number and viability were assessed using Trypan Blue staining in the Neubauer counting chamber (0.1 µL). Cell suspensions were diluted in pure medium without FCS until the desired concentration was reached. The cell implantation was performed immediately after preparation of the cell suspensions.

### Animal models and study design

Local standard operating procedures were followed for all animal handling as approved by the governing animal welfare commission. Animal Approval #0181-09 by the Landesgesundheitsamt Berlin, Germany. A total of 60 five-week-old male BALB/c mice were obtained from Charles River (Germany). All mice were injected with fourth passage K7M2 osteosarcoma cells (0.5 mL with 2.5 × 10^5^ cells per injection). The cell implantation was performed both intraperitoneally and intramuscularly into the gastrocnemius muscle in each mouse.

For the treatment, animals were divided into two arms (30 mice each) for either intravenous via central venous catheter or intraperitoneal application. In each arm, mice were either treated with 1% taurolidine (1% TRD), 2% taurolidine (2% TRD), or control (NaCl 0.9%) (10 mice per group). These dosages were chosen based on the available dose of 2% in patients and previous similar studies. The solutions were each added with 10 international units (IU) of heparin/mL. Animals in each group were treated for 7 days after cell implantation, receiving a 0.5 mL injection every 12 h containing the corresponding treatment (Table A1). Treatment with 1% TRD corresponds to 400 mg/kg taurolidine a day, and 2% TRD corresponds to 800 mg/kg taurolidine a day. After the treatment period, mice were observed for an additional 28 days, whereafter they were euthanized and autopsied for analysis.

### Evaluation of tumor growth

The tumor was exposed via median incision on the anterior aspect of the lower leg for the intramuscular tumor and via median laparotomy for the intraperitoneal tumor.

Intramuscular tumor growth was determined by measuring tumor size in all three dimensions with a precision caliper and then resecting and weighing macroscopically visible tumor mass with a precision balance. Intraperitoneal tumor growth was determined by detecting and, when possible, resecting and weighing all metastases from individual structures in the abdominal and thoracic cavities.

### Tissue fixation, paraffinization and sectioning

The resected tumor tissues were incubated in equal parts either in 10% buffered formalin solution or in zinc fixative solution to preserve tissue structure. Afterwards, tumor tissues were washed with PBS twice for 45 min and then stored in 70% ethanol at 5°C.

The fixated tumor tissues were dehydrated in a series of increasing ethanol concentrations (70%, 80%, 96%, and 100%) and embedded in paraffin blocks. After cooling, the paraffin blocks were cut into sections with a thickness of 1.5 µm and applied on Superfrost slides for further histological examination.

### Immunohistochemical staining

The histological examination of the microvessel density and the mitotic rate was performed by two different tissue staining procedures with one positive and one negative control each. Immunohistochemical staining of platelet endothelial cell adhesion molecule 1 (PECAM 1, CD31) was performed on zinc-fixed tissue sections to assess the microvessel density of the tissue samples. Additionally, immunohistochemical staining with bromodeoxyuridine (BrdU) was performed on tissue sections fixed in formalin to determine the mitotic rate. For this purpose, 50 mM (0.1 mg/g = 6.5 µL/g) BrdU was injected into the abdominal cavity of the animals two hours before sacrifice. All sections were counterstained with hematoxylin-eosin (Carl Roth GmbH, Karlsruhe) to assess tumor cell morphology, necrosis, and tumor vitality and evaluated by a trained pathologist.

In preparation for tissue staining, tissue sections were deparaffinized in xylene for 10 min three times, rehydrated in a series of decreasing ethanol concentrations (100%, 96%, 90%, 80%, and 70%), and rinsed in ultra-pure water. Then, tissue sections were washed with PBS-Tween 1% (PBST) for antigen retrieval. Tissue slides intended for BrdU staining were additionally boiled in a citrated buffer solution for 5 min. Endogenous biotin was blocked using avidin (Dako, USA). Sections were incubated overnight at 4°C with primary rat anti-CD31 (BD Pharmingen; 1:50 dilution) or rat anti-BrdU (AbD Serotec; 1:100 dilution) antibodies (both diluted in Tris-HCL buffer). Sections were washed three times in PBS-Tween 1% for 5 min and incubated with the biotinylated secondary mouse anti-rat antibody (Dako, USA; 1:200 dilution in Tris-HCL buffer) for 30 min. Sections were washed three times with PBST and incubated in previously prepared Avidin/Biotinylated enzyme complex (ABC-Vectastain AK 5004; ratio 1:1; Vector Laboratories Inc., USA; 1:50 dilution in PBS) for 30 min. Incubation was terminated with three washing steps in PBST. Finally, the sections were incubated with the enzyme substrate Fast Red (Dako, USA) for 30 min for CD31 staining and 18 min for BrdU staining both determined with doubly distilled water. Analysis was guided by a trained pathologist.

### Microvessel density and mitotic rate

Microvessel density (CD31 staining) was determined by counting “hot spots” (areas in the tumor with high vessel density) at 400× magnification based on prior studies [24,25]. Twenty fields of view were counted per sample. After determining the field of view (diameter (d) of the visual field in mm, field number of the ocular (=25), scale number of the objective (=40)), the field of view area in mm² (=0.31 mm²) was calculated using the formula for the area of a circle (A = r²* π = (1/2d)²* π). Results of the field of view count (=0.31 mm²) were extrapolated to 1 mm². Evaluation of the mitotic rate/1 mm² (BrdU staining) followed the same procedure.

### Statistical analysis

Statistical analysis was performed with SPSS version 29.0 (IBM Corp., Armonk, NY, USA). The Mann-Whitney-U-Test was used for orientation to detect differences between the groups. Pearson’s test was used to assess correlation between microvessel density, mitotic rate, and tumor weight. Test results were considered statistically significant at the level of *p* ≤ 0.05.

## Results

### Animal inclusion

Animals were included in the analysis if they completed corresponding treatment schedules and if they were in stable condition during the follow-up period. In total, 42 out of the 60 original animals were included in the analysis. Seven animals were excluded due to central venous catheter dysfunction or because they were randomly selected for training in handling the central venous catheter (Table 1). Another 11 animals were excluded due to mortality, which was caused by per interventional vagotonic reactions or intraperitoneal hemorrhage, or because animals had to be prematurely sacrificed due to reduced general conditions per the local animal welfare commission (Table 1). Since seven out of the ten animals in the control group in the intravenous treatment arm were excluded, a combined control group from both the animals in the intravenous and intraperitoneal treatment arm is used for the analysis in the intravenous treatment arm. The analysis in the intraperitoneal treatment arm was performed with only the 10 control animals who were treated with NaCl 0.9% intraperitoneal.

**Table 1:** Included animals and reasons for exclusion

Treatment group (n included animals)	Reasons for exclusion (n)
NaCl 0.9% (13)	CVC dysfunction (2)
	Experimental animals for CVC handling (3)
	Premature death due to reduced general condition (2)
IV 1% TRD (9)	Death during IV injection due to vagotonic reaction (1)
IV 2% TRD (6)	Death during IV injection due to vagotonic reaction (2)
	CVC dysfunction (2)
IP 1% TRD (10)	NA
IP 2% TRD (4)	Premature death due to reduced general condition (5)
	Arterial intraperitoneal bleeding (1)

### Animal weight

**Figure 1:**
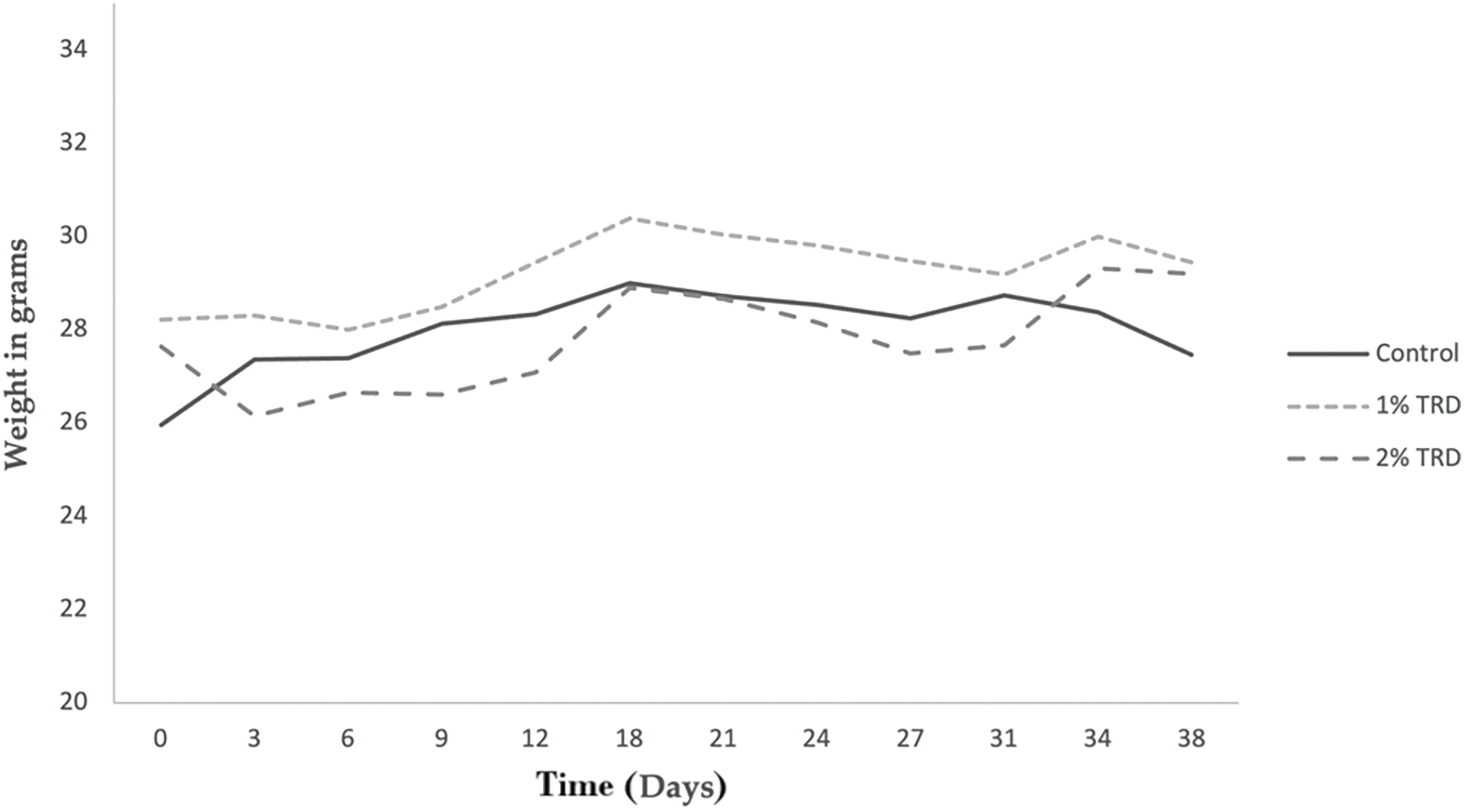
Weight progression in animals in the intravenous treatment arm.

**Figure 2:**
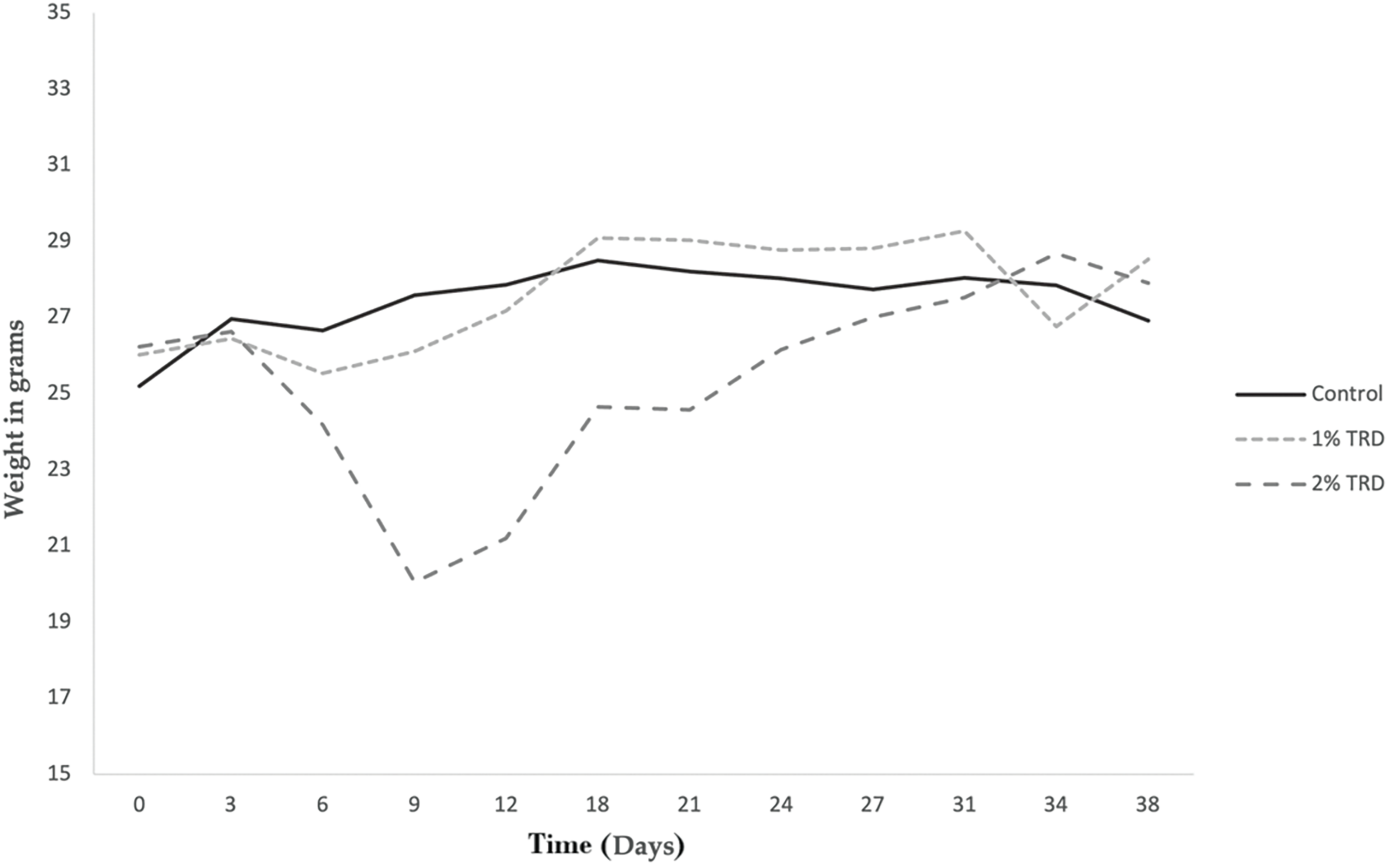
Weight progression in animals in the intraperitoneal treatment arm.

Animals included in the study had an initial median weight of 25.5 g (22.7–29.1 g). There were no statistically significant differences in initial weight between treatment groups. The weight of the animals was measured every 3 days during both the treatment period (days 1–7) and the follow-up period (days 8–35). Fig. 1 shows the mean weight progression in the intravenous treatment arm. No clear trends are seen. The mean weight progression in the intraperitoneal treatment arm is shown in Fig. 2. In the 2% TRD group, a clear weight reduction is seen after 3 days and with a peak after 9 days. The weight regains to baseline after 31 days. No trends are seen in the 1% TRD group.

### Tumor growth

In the intravenous administration arm, there were no significant differences in intramuscular or intraperitoneal tumor weight between treatment groups (Table 2). In the intraperitoneal administration arm, the intraperitoneal tumor weight was significantly lower in both 1% and 2% TRD treatment groups compared to the control (*p* = 0.006 and *p* = 0.008, respectively; Table 2). There was no significant difference in intraperitoneal tumor weight between 1% and 2% TRD treatment groups in the intraperitoneal administration arm (*p* = 0.60). There were no significant differences in intramuscular tumor weight between treatment groups in the intraperitoneal administration arm.

**Table 2:** Tumor weight

Treatment arm	Treatment group	Median tumor weight (interquartile range)	*p*-value
	*Intramuscular tumor growth*		
Intraperitoneal	NaCl 0.9%	1690 mg (2775)	
	1% TRD	3630 mg (3050)	0.50
	2% TRD	3625 mg (863)	0.19
Intravenous	NaCl 0.9%	1820 mg (2055)	
	1% TRD	2800 mg (3220)	0.60
	2% TRD	1190 mg (2358)	0.47
	*Intraperitoneal tumor growth*		
Intraperitoneal	NaCl 0.9%	700 mg (2104)	
	1% TRD	0 mg (10)	**0.006**
	2% TRD	0 mg (0)	**0.008**
Intravenous	NaCl 0.9%	580 mg (1920)	
	1% TRD	830 mg (1417)	0.60
	2% TRD	215 mg (1470)	0.28

The Man-Whitney U test was used to compare the control group (NaCl 0.9%) and the TRD treatment groups.

### Microvessel density and mitotic rate

**Figure 3:**
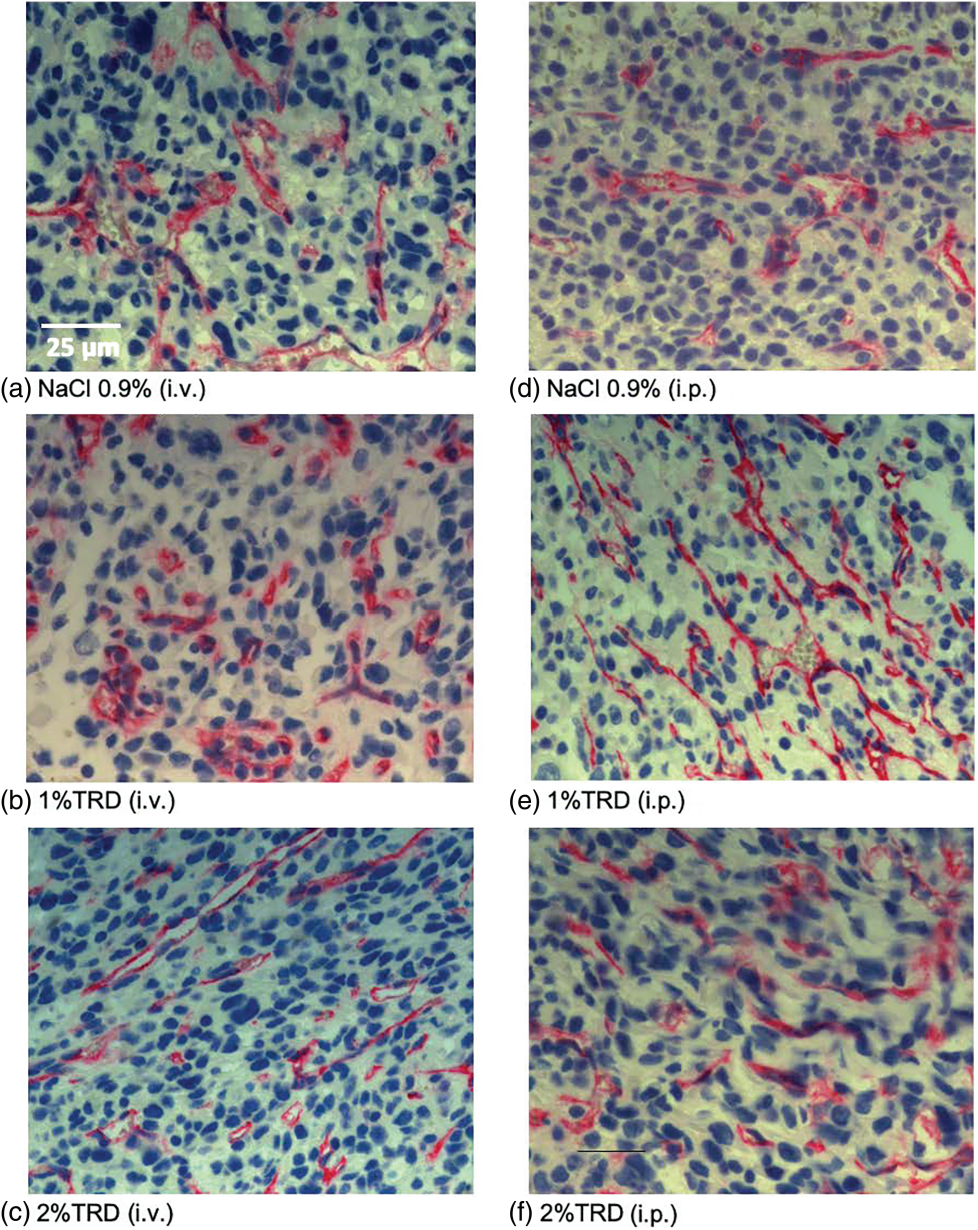
Representative CD31 staining.

**Figure 4:**
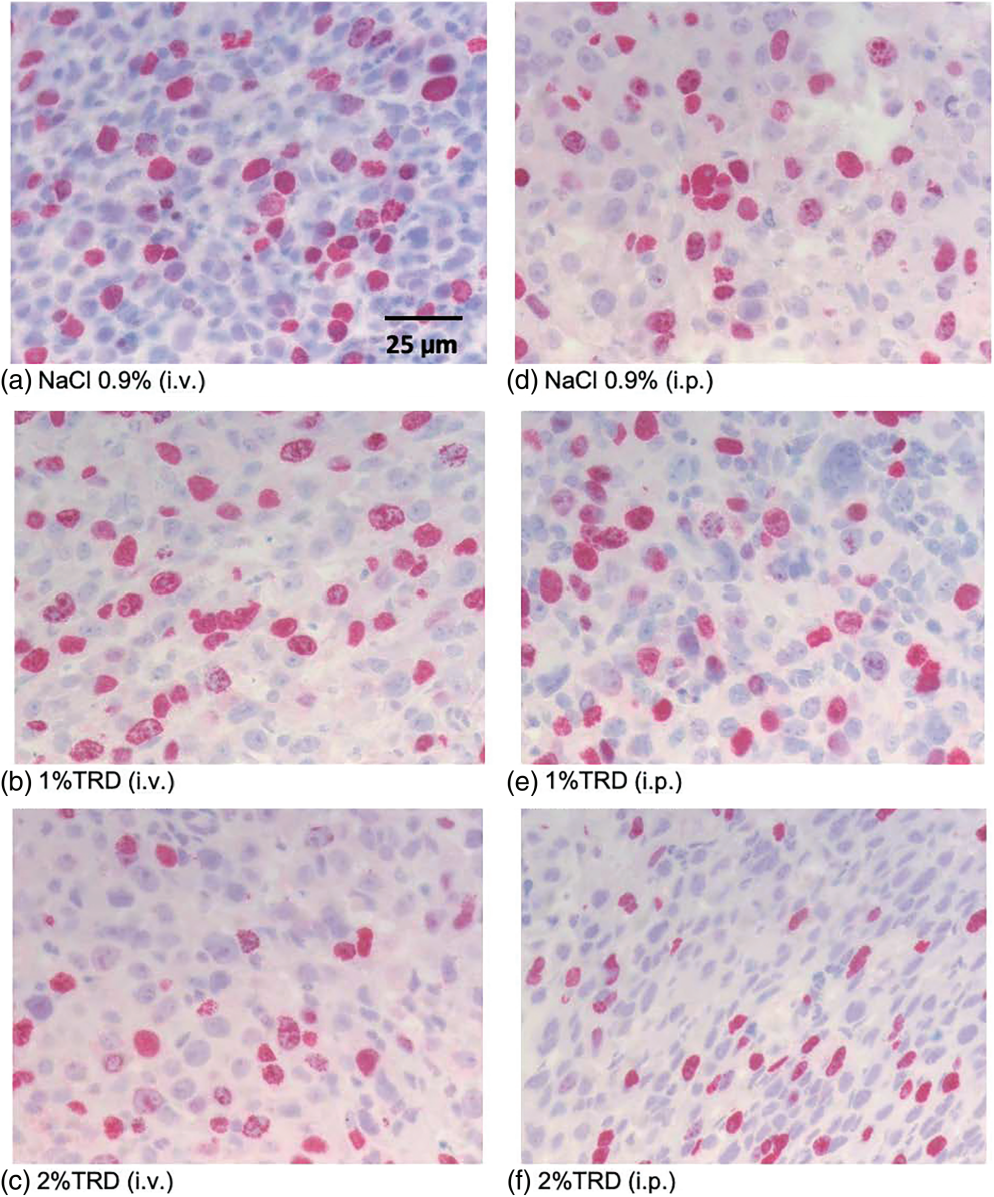
Representative BrdU staining.

As a marker of angiogenesis given its importance to the metastasis of osteosarcoma and the established effects of taurolidine on angiogenic processes, microvessel density was visualized by CD31 staining, and microvessel counts were determined across administration arms and treatment groups (Fig. 3). There were no significant differences in microvessel counts for 1% or 2% TRD treatment groups compared to control for the intravenous and intraperitoneal administration arms (Table 3). Next, mitotic activity was visualized by BrdU staining as a marker of tumor activity, and mitotic rates were determined across administration arms and treatment groups (Fig. 4). There were no significant differences in mitotic rates for 1% or 2% TRD treatment groups compared to control for the intravenous and intraperitoneal administration arms (Table 3).

**Table 3:** Microvessel count and mitotic rate

Treatment group	Mean vessel count per mm²	*p*-value	Mean mitoses/mm^2^	*p*-value
*Intravenous treatment arm*				
NaCl 0.9%	66.1		94.0	
1%	73.3	0.41	82.6	1.00
2%	61.9	0.92	50.2	0.20
*Intraperitoneal treatment arm*				
NaCl 0.9%	70.5		94.0	
1%	65.8	1.00	94.5	1.00
2%	64.1	0.76	115.6	0.20

The Man-Whitney U test was used to compare the control group (NaCl 0.9%) with the TRD treatment groups.

### Side effects

Intravenous administration of TRD-induced vagotonic reactions leading to respiratory depression in multiple animals. In most animals, this was rapidly reversible. However, in three animals this resulted in death. In addition, a transient dose- and route of administration-dependent weight reduction of animals was observed in TRD groups, accompanied by diarrhea (Figs. 1 and 2). This observation was most pronounced in the group that received 2% TRD by intraperitoneal administration. Both the weight loss and diarrhea resolved during the follow-up period.

## Discussion

In this study, we investigated the antitumor and antiangiogenic effects of intravenous or intraperitoneal taurolidine on intramuscular and intraperitoneal osteosarcomas in mice. A significant reduction in tumor growth was seen for intraperitoneal osteosarcomas after treatment with 1% or 2% TRD by intraperitoneal administration. No effect was observed for intramuscular tumors after treatment with TRD by intraperitoneal or intravenous administration, and no effect on intraperitoneal tumors was seen for TRD when administered intravenously. We observed no antiangiogenic effects of TRD treatment as assessed by microvessel density and mitotic rate.

The antineoplastic effect of TRD has been demonstrated in multiple *in vitro* and *in vivo* studies [18,26–30] using colorectal, pancreatic, lung, glioma, and melanoma cancer cell lines [31]. TRD has also been shown to inhibit the growth of canine osteosarcoma and 10 different human osteosarcoma cell lines *in vitro* [32,33]. The effect of TRD on osteosarcoma cells *in vivo* has been evaluated in murine models based on K7M2 and LM8 cell lines that had demonstrated antineoplastic effects *in vitro* [34]. In both murine model studies, no effect on the primary tumor was observed after treatment with taurolidine by intraperitoneal administration, and a higher rate of lung and liver metastases in LM8 models was observed after treatment with taurolidine. In contrast, we found in the K7M2 murine model that intraperitoneal treatment with taurolidine reduced intraperitoneal tumor weight. Our study differs in many aspects, including the method of K7M2 tumor cell introduction, TRD dosage, and treatment duration, making it challenging to compare the results directly.

In addition, several prior studies demonstrated the antineoplastic effects of TRD when administered intravenously for the treatment of other tumor types [35–37]. However, we observed no effect of TRD on tumor growth when administered intravenously. A possible explanation for this observation could relate to osteosarcoma, as none of the prior studies involved mesenchymal malignancies such as osteosarcoma. Another potential cause of this observation could be that the dose of TRD we utilized was not high enough to observe effects by intravenous administration, as a prior study demonstrating intravenous efficacy of TRD on colon carcinomas utilized a 3% TRD solution and failed to show effects at 1% or 2% [38]. The difference within antitumor effect within the intraperitoneal and intravenous treatment groups could be explained by the fact that in the intraperitoneal treatment group TRD is directly administrated to the intraperitoneal tumors, while in the intravenous treatment group multiple barriers must be crossed, and likely a lower dose TRD reached the tumors.

Furthermore, we observed no antineoplastic effects of TRD on intramuscular osteosarcomas despite observed reductions in intraperitoneal tumors. Previous studies also show mixed results for effects of TRD tumor growth by location. While several studies demonstrated an effect of intraperitoneal TRD treatment on growth across anatomical locations, several other studies showed no effect [37,39–41]. It is unclear whether these observations are due to treatment dose and duration, method of tumor introduction, origin or tumor cells, and/or other factors.

Two main side effects were observed during this study. At first, vagotonic reactions associated with respiratory depression occurred after intravenous administration of TRD, which led to the death of three animals. This phenomenon has been described previously and appears to be caused by rapid intravenous injection of TRD [38]. In addition, a temporary significant weight loss was observed after treatment with 2% TRD by intraperitoneal administration. As a result, five animals in this treatment group had to be euthanized prematurely. An earlier study on osteosarcoma mice models also observed reduced water and food intake resulting in weight loss after administration of a high dose of TRD [34]. They also report severe liver toxicity. However, in a study in dogs with osteosarcomas no signs of liver toxicity were observed [42]. A potential explanation for the observed toxicity is the degradation of TRD to the toxic formic acid and formaldehyde [43,44].

Therapies that inhibit VEGF or angiogenesis have proven to be promising treatments for osteosarcoma, and since TRD was previously shown to inhibit the VEGF synthesis, TRD may be a promising agent [38,45–47]. In this study, however, no effects of TRD on microvessel density or mitotic rate were observed. A possible explanation could be that microvessel density and mitotic rate are not related to VEGF synthesis or that CD31 and BrdU staining are not reflective of VEGF synthesis. This theory is supported by a study by Rossi et al. [48], in which a reduction in VEGF synthesis was seen without impact on microvessel density as evaluated with CD31 staining. It is also possible that the inhibition of tumor growth observed in the osteosarcoma mice models is based on other pathways then the inhibition of VEGF synthesis. Earlier studies observed that TRD induces apoptosis in osteosarcoma cells and that it inhibits the adhesions of osteosarcoma cells [32,33]. Both of these effects were dose dependent.

Before TRD could be added to the treatment of patients with osteosarcomas more research is warranted, also to investigate the effect of TRD within the current treatment strategy. The addition of TRD to the current treatment strategy could hopefully lead to a reduction in dose of the currently used chemotherapies methotrexate, doxorubicin, and cisplatin. All these agents are associated with severe adverse events. An earlier *in vitro* study on osteosarcoma cell lines showed that TRD has the potential to improve the cytotoxicity of doxorubicin [33]. A study on osteosarcoma in dogs observed that taurolidine possible intensifies the toxicity of doxorubicin and carboplatin [42]. However, this has not been validated in a large trial. Our study only showed an effect of intraperitoneal treatment with TRD. Ideally, the agent would be administrated to patients intravenously, however, it would also be possible to administrate the agent intraperitoneally. Hypothermic intraperitoneal chemotherapy (HIPEC) is widely used for patients with gynecological cancers or colorectal peritoneal metastases [49,50].

A limitation of this study is the small sample size due to the exclusion of 18 out of the 60 animals. As a result, the control group in the intravenous treatment arm needed to be combined for the analysis. This could have influenced the results since the administration route itself could potentially have an influence on the mice models. However, despite that a clear antineoplastic effect on intraperitoneal osteosarcomas is observed after intraperitoneal administration of TRD. In addition, clear toxicity of treatment with 2% TRD intraperitoneal is observed, shown by the high premature death rate due to reduced general condition, and the weight loss trend in this treatment group.

## Conclusion

Intraperitoneal administration of taurolidine had an antineoplastic effect on intraperitoneal osteosarcomas. Due to adverse reactions, a dose of 400 mg/kg is likely preferable compared to a dose of 800 mg/kg for murine studies. No antineoplastic effects were observed on intramuscular tumors or for intravenous administration of taurolidine. Microvessel density and the mitotic rate were not different between treatment groups in this study. In conclusion, there is no evidence of antiangiogenic effects, and the observed antitumor effects of taurolidine on osteosarcomas in mice are limited. Moreover, its toxic profile grants further evaluation. Given these observations, further research is necessary to refine the use of taurolidine in osteosarcoma treatment.

## Data Availability

The data presented in this study are available on request from the corresponding author. The data are not publicly available due to ethical restrictions.

## Appendix

**Table A1:** Treatment arms and groups

Treatment arms	Treatment groups	n
Intravenous administration	NaCl 0.9% (control)	10
	1% TRD (400 mg/kg per day)	10
	2% TRD (800 mg/kg per day)	10
Intraperitoneal administration	NaCl 0.9% (control)	10
	1% TRD (400 mg/kg per day)	10
	2% TRD (800 mg/kg per day)	10
